# Isomaltulose Exhibits Prebiotic Activity, and Modulates Gut Microbiota, the Production of Short Chain Fatty Acids, and Secondary Bile Acids in Rats

**DOI:** 10.3390/molecules26092464

**Published:** 2021-04-23

**Authors:** Zhan-Dong Yang, Yi-Shan Guo, Jun-Sheng Huang, Ya-Fei Gao, Fei Peng, Ri-Yi Xu, Hui-Hui Su, Ping-Jun Zhang

**Affiliations:** 1 School of Pharmaceutical Sciences, Sun Yat-sen University, Guangzhou 510006, China; yangzhd5@mail2.sysu.edu.cn; 2 Guangdong Engineering Lab of High Value Utilization of Biomass, Institute of Bioengineering, Guangdong Academy of Sciences, Guangzhou 510316, China; yishan.guo@foxmail.com (Y.-S.G.); huangjunsheng2020@foxmail.com (J.-S.H.); csicgao@foxmail.com (Y.-F.G.); scutriyal@iCloud.com (R.-Y.X.); 3 School of Food Science and Engineering, Nanchang University, Nanchang 330000, China; pengf0129@foxmail.com

**Keywords:** isomaltulose, prebiotics, gut microbiota, secondary bile acids, short chain fatty acids

## Abstract

In vitro experiments have indicated prebiotic activity of isomaltulose, which stimulates the growth of probiotics and the production of short chain fatty acids (SCFAs). However, the absence of in vivo trials undermines these results. This study aims to investigate the effect of isomaltulose on composition and functionality of gut microbiota in rats. Twelve Sprague–Dawley rats were divided into two groups: the IsoMTL group was given free access to water containing 10% isomaltulose (*w/w*), and the control group was treated with normal water for five weeks. Moreover, 16S rRNA sequencing showed that ingestion of isomaltulose increased the abundances of beneficial microbiota, such as *Faecalibacterium* and *Phascolarctobacterium*, and decreased levels of pathogens, including *Shuttleworthia*. Bacterial functional prediction showed that isomaltulose affected gut microbial functionalities, including secondary bile acid biosynthesis. Targeted metabolomics demonstrated that isomaltulose supplementation enhanced cholic acid concentration, and reduced levels of lithocholic acid, deoxycholic acid, dehydrocholic acid, and hyodeoxycholic acid. Moreover, the concentrations of propionate and butyrate were elevated in the rats administered with isomaltulose. This work suggests that isomaltulose modulates gut microbiota and the production of SCFAs and secondary bile acids in rats, which provides a scientific basis on the use of isomaltulose as a prebiotic.

## 1. Introduction

Gut microbiota plays an important role in the host diet, nutrient production and absorption, metabolism, and maintenance of the immune system [1]. A large quantity of evidence has demonstrated that dysbiosis of intestinal bacteria is closely related to several diseases, including irritable bowel syndrome, diverticular disease and celiac disease, and type 2 diabetes mellitus [2,3,4,5]. It is, thereby, a crucial strategy to improve host wellness is the maintenance of gut microbial homeostasis. Prebiotics are effective microbiota-management tool; a prebiotic is described as a substrate that is selectively utilized by host microorganisms conferring a health benefit [6,7]. Inulin, galactooligosaccharides (GOS) and fructooligosaccharides (FOS) are the most prevalently used commercial prebiotic ingredients worldwide. These prebiotics have exhibited crucial efficiency in counteracting diseases [8,9]. For one, prebiotics can be utilized by beneficial microbiota and support their growth in the intestine [7]. The latter forcefully competes for nutrients and adhesion sites with opportunistic pathogens [10]. In addition, beneficial microorganisms may interact with host cells, which contributes to the intestinal barrier and immune functions [9].

Furthermore, prebiotics can affect the composition and proportion of gut microbial metabolites, which may be involved in the occurrence and development of several diseases including depression, colorectal cancer, obesity, and type 2 diabetes [11]. Short chain fatty acids (SCFAs), including acetic acid, propionic acid, and butyric acid, are an extremely important class of these metabolites. SCFAs play a crucial role in the maintenance of the host–gut homeostasis and health status. The mechanisms underlying SCFAs may be: (a) SCFAs decrease inflammatory responses, modulate the immune system, and induce apoptosis of cancer cells; (b) SCFAs, in turn, regulate gut microbial composition by inhibiting the conditioned-pathogen growth; (c) emerging research shows that SCFAs may promote the gut–brain communication, which thereby affects multiple neurochemical pathways [2]. Another important type of metabolites are secondary bile acids (SBAs) from the conversion of primary bile acids by gut microbiota. An increasing number of studies show that SBAs may exhibit diverse roles in host physiology [12]. In particular, deoxycholic acid (DCA) and lithocholic acid (LCA) have demonstrated to suppress the proliferation of *Clostridium difficile*, induce hepatocellular carcinogenesis, and regulate metabolic and immune responses [13]. Moreover, DCA and LCA can be further modified into additional SBAs by intestinal microbiota, i.e., hyodeoxycholic acid (HDCA). HDCA is suspected to have high cytotoxicity and anaphylactic roles [14,15,16]. Whereas several studies reported that HDCA prevents gallstones and atherosclerosis [17,18].

Therefore, prebiotics are the necessary nutrients for the maintenance of human health and counteracting diseases. Isomaltulose naturally occurs in honey and sugar cane juice [19]. Due to that, it exhibits many excellent properties, including similar organoleptic quality and processability to sucrose, low glycemic index, and non-cariogenicity; isomaltulose has been extensively used as sugar alternative in the food industry [20]. In our previous study, isomaltulose promoted the proliferation of selected probiotics [21]. Moreover, it affected the production of SCFAs in coculture with specific probiotic strains. Another in vitro human colonic model research demonstrated that isomaltulose reduced the ratio of *Firmicutes* to *Bacteroidetes*, and increased the level of acetic and butyric acids [22]. Therefore, these in vitro experiments exhibit that isomaltulose may have prebiotic activity. However, there is a substantial difference in intestinal microbial composition between in vitro and in vivo experiments. The present study aimed to explore the modulation effect of isomaltulose on gut microbiota in rats. In addition, we investigated changes in the production of SCFAs and SBAs by dietary isomaltulose. This study may provide a scientific basis of the prebiotic activity of isomaltulose and, thus, contribute toward boosting its industrial application as a prebiotic ingredient.

## 2. Results

### 2.1. Isomaltulose Affects Physiological and Biochemical Parameters in Rats

**Figure 1:**
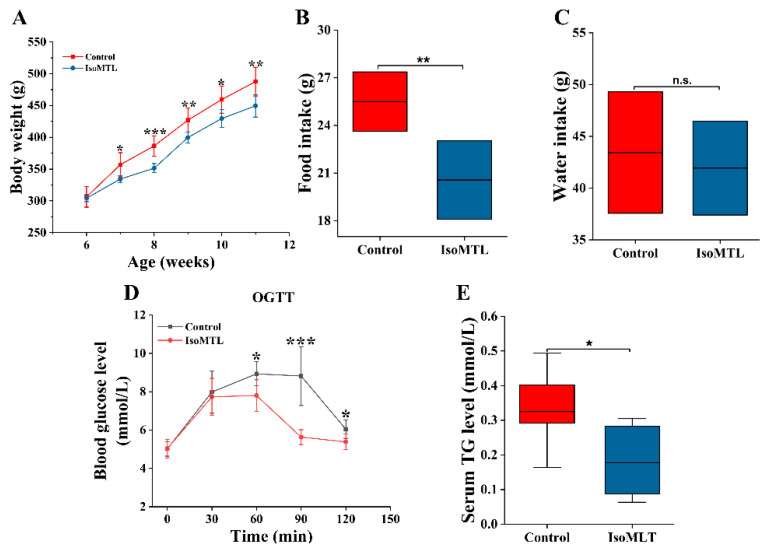
Dietary isomaltulose affects physiological and biochemical parameters in rats. (**A**) Body weight. (**B**) Food intake and (**C**) water intake. (**D**) The effect on oral glucose tolerance (OGTT). (**E**) The effect on triglyceride (TG). n.s. refers to no significant difference between two groups. * Represents differences between two groups (* *p* < 0.05, ** *p* < 0.01, *** *p* < 0.001).

During the entire experiments, the body weight of each rat was measured once each week. As shown in Figure 1A, there was significantly lower body weight in the group with isomaltulose compared to the control group (*p* < 0.05). The daily average food intake was also less in the IsoMTL group at the end of this experiment (*p* < 0.01), whereas the water intake showed no significant difference between two groups (Figure 1B,C). Subsequently, we determined the effect of dietary isomaltulose on glycolipid metabolism in rats. As indicated in Figure S1A–C, the levels of fasting blood glucose (FBG), fasting serum insulin (FSIns), and homeostasis model assessment of insulin resistance (HOMA-IR) showed no significant difference between rats with and without isomaltulose. However, the oral glucose tolerance test (OGTT) revealed lower blood glucose levels and faster recovery to normal levels in rats consuming isomaltulose (Figure 1D). Moreover, dietary isomaltulose significantly reduced serum triglyceride (TG) levels (*p* < 0.05, Figure 1E), while it did not affect serum levels of total cholesterol (TCHO), low-density lipoprotein (LDL), and high-density lipoprotein (HDL) (Figure S1D–F). Serum level of lipopolysaccharide (LPS) showed no significant difference between two groups (*p* > 0.05, Figure S2). In addition, we observed no histologic lesion in the livers or colons in the rats fed with isomaltulose when compared to the control ones (Figure S3).

### 2.2. Isomaltulose Regulates the Diversity of Gut Microbiota

**Figure 2:**
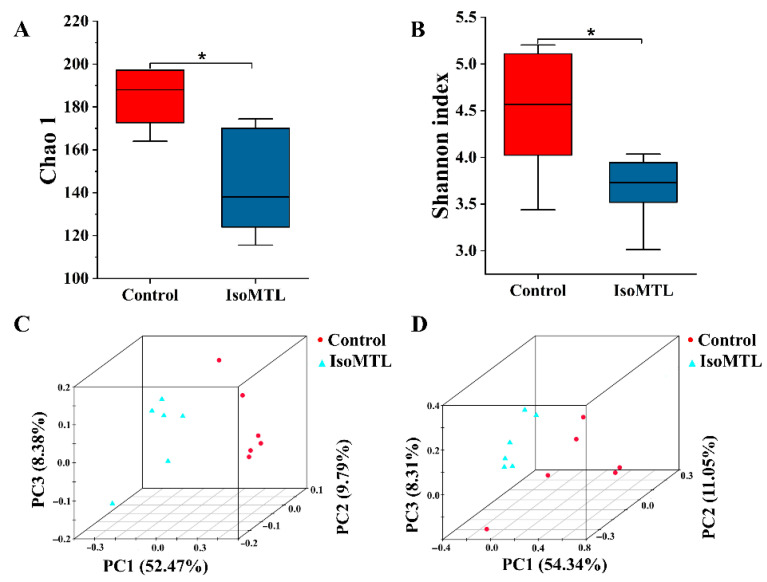
Isomaltulose affects the species diversity of gut microbiota. The alpha diversity includes the Chao 1 index (**A**) and Shannon index (**B**). The beta diversity includes the unweighted UniFrac PCoA score plot (**C**) and weighted UniFrac PCoA score plot (**D**). * Represents differences between two groups (* *p* < 0.05).

To explore the effect of isomaltulose ingestion on gut microbiota, the fecal bacterial 16S rRNA sequencing was performed using the MiSeq platform. The Chao and Shannon index values were used to evaluate species diversity and richness (α-diversity) in each microbiome sample. As shown in Figure 2A,B, the Chao and Shannon index values were decreased in the rats fed with isomaltulose when compared to the control rats (*p* < 0.05). A principal coordinate analysis (PCoA) score plot can present a visual assessment of the ecological complexity (β-diversity) of the fecal microbiome between two groups. In the present study, the unweighted and weighted PCoA score plots showed ambiguous separations between control and IsoMTL groups (Figure 2C,D). Moreover, the samples with the same color were clustered together. These results suggested that there was a species similarity within the groups.

**Figure 3:**
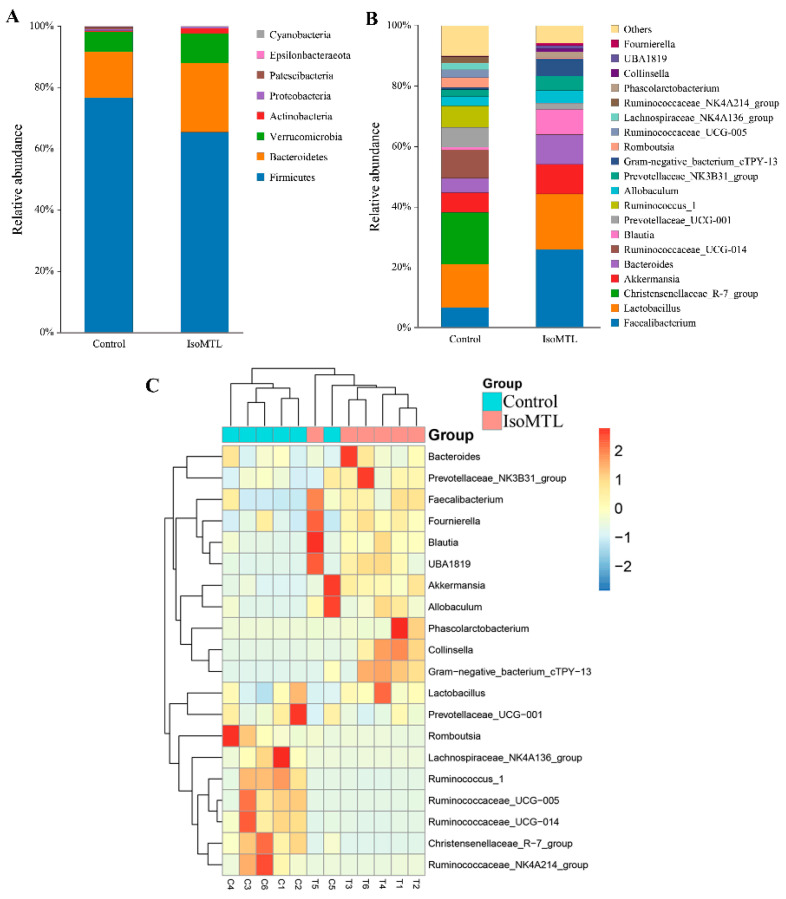
Isomaltulose regulates gut microbiota in rats. (**A**) The bacterial composition at the phylum levels between two groups. (**B**) The top 20 bacterial genera in relative abundance. (**C**) The heatmap analysis based on the abundance of the top 20 microbial genera.

The composition and structure of gut microbial populations in rats was analyzed at different taxonomic levels. At the phylum level, the top four bacterial phyla were *Firmicutes*, *Bacteroidetes*, Verrucomicrobia, and *Actinobacteria*, which accounted for 98.95% of the total OTU numbers (Figure 3A). Moreover, the relative abundance of *Firmicutes* was reduced, whereas the relative level of *Bacteroidetes*, *Verrucomicrobia,* and *Actinobacteria* increased by dietary isomaltulose (Figure 3A). At the genus level, the IsoMTL showed high abundance of *Faecalibacterium*, *Lactobacillus*, *Akkermansia*, *Bacteroides,* and *Blautia*, whereas the control group revealed high abundance of *Christensenellaceae_R-7_group*, *Lactobacillus*, *Faecalibacterium*, *Ruminococcaceae_UCG-00*1, and *Akkermansia* (Figure 3B). To investigate specific patterns along with each microbiota and sample grouping, a heatmap profiling was performed based on relative abundance of the top 20 bacterial genera. As illustrated in Figure 3C, two distinct clusters were observed. *Bacteroides*, *Prevotellaceaae_NK3B31_group*, *Faecalibacterium*, *Fournierella*, *Blautia*, *UBA1819*, *Akkermansia*, *Allobaculum*, *Phascolarctobacterium*, *Collinsella*, *Gram-negative_bacterium_Ctpy-13,* and *Lactobacillus* were mainly clustered in the IsoMTL group. Whereas *Prevotellaceae_UCG-001*, *Romboutsia*, *Lachnospiraceae_NK4A136_group*, *Ruminococcus_1*, *Ruminococceae_UCG-005*, *Ruminococceae_UCG-014*, *Christensenellaceae_R-7_group,* and *Ruminococceae_NK4A214_group* were gathered in control group. Therefore, our data demonstrated that the consumption of isomaltulose tended to affect the composition and structure of gut microbial populations in healthy rats.

### 2.3. Significantly Altered Gut Microbiota at Each Taxonomic Levels by Isomaltulose

**Figure 4:**
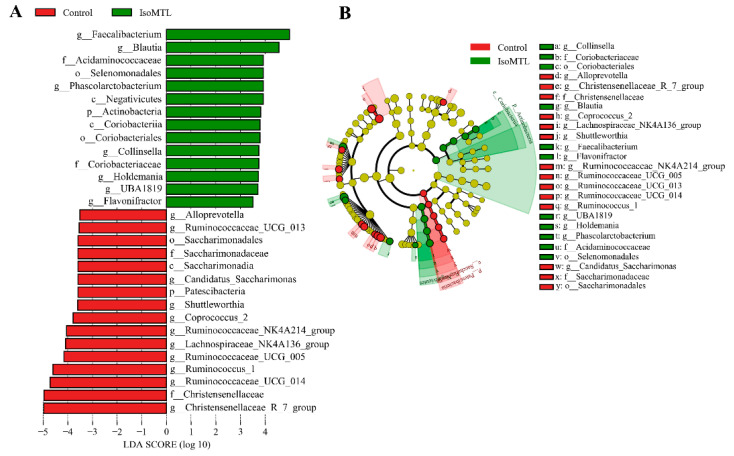
The significant altered microbiota at different taxonomic levels. (**A**) Significantly different bacterial species with LDA score log 10 ≥ 3.5 and *p* < 0.05 from phylum to genus. (**B**) Taxonomic cladogram obtained through LEfSe profiling based on 16S rRNA sequencing data.

**Figure 5:**
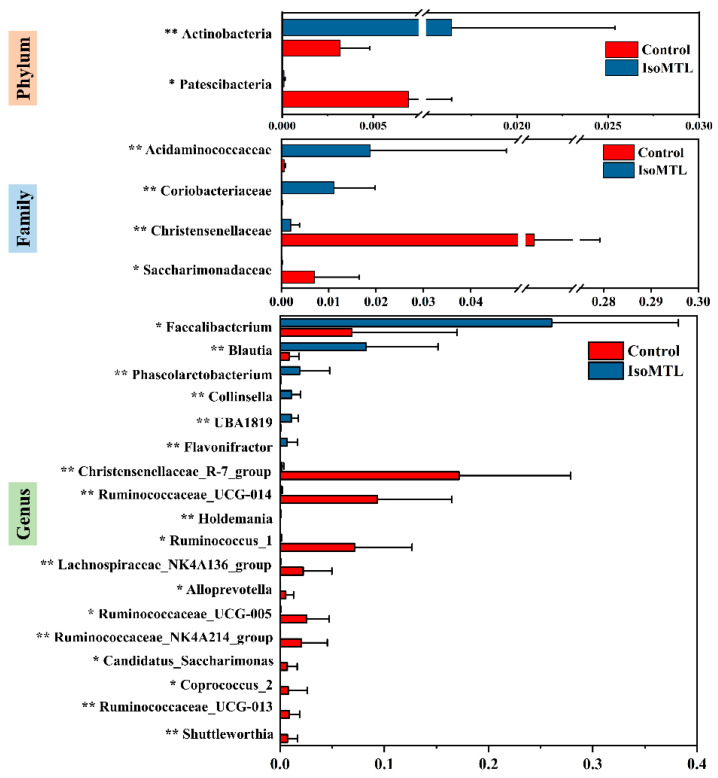
The significantly altered microbiota at phylum, family, and genus levels by LEfSe analysis. * represents the differences between control and IsoMTL rats (* *p* < 0.05, ** *p* < 0.01).

To further identify significantly affected intestinal bacterial communities at each taxonomic level from phyla to genera, we carried out the LEfSe analysis. The threshold value of LDA score log 10 was set as not less than 3.5. As shown in Figure 4A,B, A total of 30 taxa exhibited an obvious difference between the control group and IsoMTL group. At the phylum level, the relative level of *Actinobacteria* was significantly increased in the rats fed with isomaltulose (*p* < 0.01), whereas the relative abundance of *Patescibacteria* was markedly higher in the control group (*p* < 0.05). In comparison with the control rats, the abundant class *Negativicutes* and *Coriobacteriia* were observably enhanced (*p* < 0.01), while *Saccharimonadia* was significantly lowered by dietary isomaltulose (*p* < 0.05). The relative abundance of bacterial orders *Selenomonadales* and *Coriobacteriales* were notably elevated (*p* < 0.01), but *Saccharimonadales* was significantly lower in the IsoMTL group (*p* < 0.05). A total of four microbial family were altered in the relative abundance between two groups. The consumption of isomaltulose sharply elevated the abundance of *Acidaminococcaceae* and *Coriobacteriaceae* (*p* < 0.01), but reduced the level of *Christensenellaceae* and *Saccharimonadaceae* (*p* < 0.01). Moreover, many bacterial genera showed significant difference between the control group and IsoMTL group. Among these microbiota, seven taxa, *Faecalibacterium*, *Blautia*, *Phascolarctobacterium*, *Collinsella*, *Holdemania*, *UBA1819* and *Flavonifractor*, were dramatically enriched in IsoMTL group (*p* < 0.05), but eleven bacterial genera, *Alloprevotella*, *Ruminococcaceae_UCG_013*, *Candidatus_Saccharimonas*, *Shuttleworthia*, *Coprococcus_2*, *Ruminococcaceae_NK4A214_group*, *Lachnospiraceae_NK4A136_group*, *Ruminococcaceae_UCG_005*, *Ruminococcus_1*, *Ruminococcaceae_UCG_014,* and *Christensenellaceae_R-7_group*, were significantly decreased by isomaltulose supplementation (*p* < 0.05). Further, the changes in different taxonomic levels (phylum, family, and genus) were presented in Figure 5.

### 2.4. Predicted Functional Composition of Gut Microbiota Related to Dietary Isomaltulose

**Figure 6:**
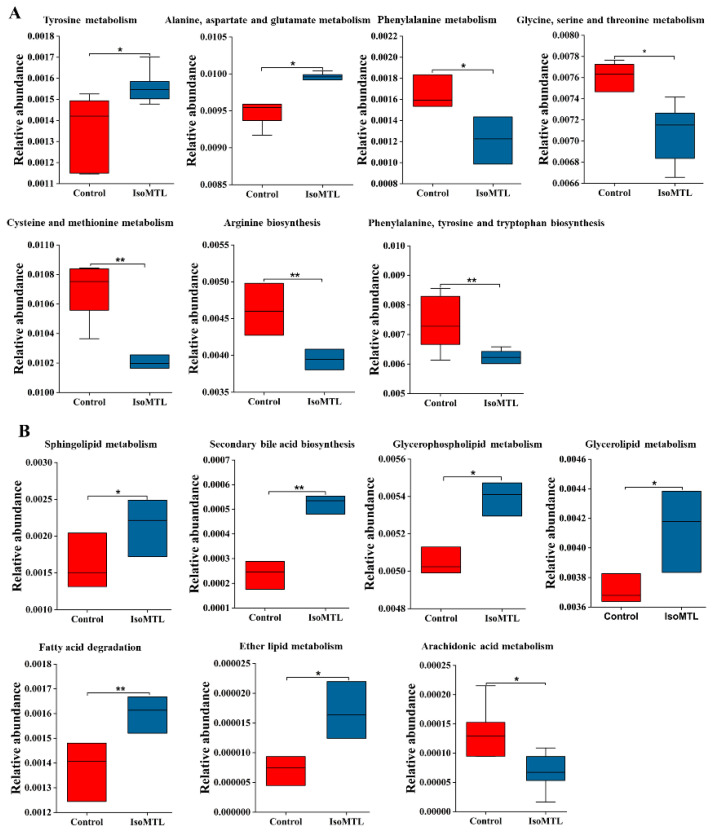
Predicted intestinal microbial functions by PICRUSt2 using 16S rRNA sequencing data. (**A**) Amino acid metabolism. (**B**) Lipid metabolism. * Represents the differences between control and IsoMTL rats (* *p* < 0.05, ** *p* < 0.01).

**Figure 7:**
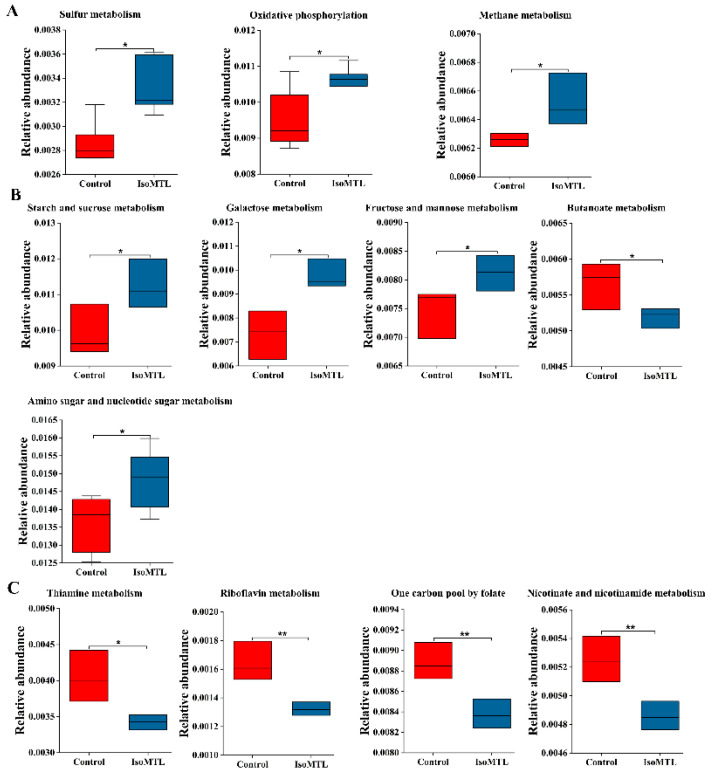
Predicted intestinal microbial functions by PICRUSt2 using 16S rRNA sequencing data. (**A**) Energy metabolism. (**B**) Carbohydrate metabolism. (**C**) The biosynthesis of cofactors and vitamins. * represents the differences between control and IsoMTL rats (* *p* < 0.05, ** *p* < 0.01).

Gut microbiota has various metabolic functions and activities [23]. Moreover, an in-depth understanding of intestinal microbial functionality may contribute to elaborating the potential effect of isomaltulose supplementation on host health. Thus, we predicted the functional composition of gut microbiota using PICRUSt2 based on the 16S rRNA sequencing data. There were a total of 254 metabolic functions enriched, including carbohydrate metabolism, amino acid metabolism, lipid metabolism, energy metabolism, and metabolism of cofactors and vitamins (Table S1). Among seven amino acid metabolic pathways, tyrosine, alanine, aspartate and glutamate metabolisms were increased, while metabolisms of phenylalanine, glycine, serine, threonine, cysteine, and methionine, and biosynthesis of arginine, phenylalanine, tyrosine, and tryptophan were reduced in the IsoMTL group (Figure 6A). Five lipid metabolism pathways, including secondary bile acid biosynthesis showed higher abundance, whereas arachidonic acid metabolism was significantly reduced in the IsoMTL group (Figure 6B). Three energy metabolism, sulfur, oxidative phosphorylation, and methane metabolisms were enriched in the IsoMTL group (Figure 7A). Four carbohydrate metabolic pathways, such as starch and sucrose, galactose, fructose and mannose, and amino sugar and nucleotide sugar metabolisms, were elevated, but butanoate metabolism decreased in the rats fed with isomaltulose (Figure 7B). In addition, four cofactor and vitamin metabolism pathways were significantly reduced in the IsoMTL group (Figure 7C). These results indicated that dietary isomaltulose could modulate the gut microbial functionalities.

### 2.5. Isomaltulose Affects the Fecal Levels of Secondary Bile Acids

**Figure 8:**
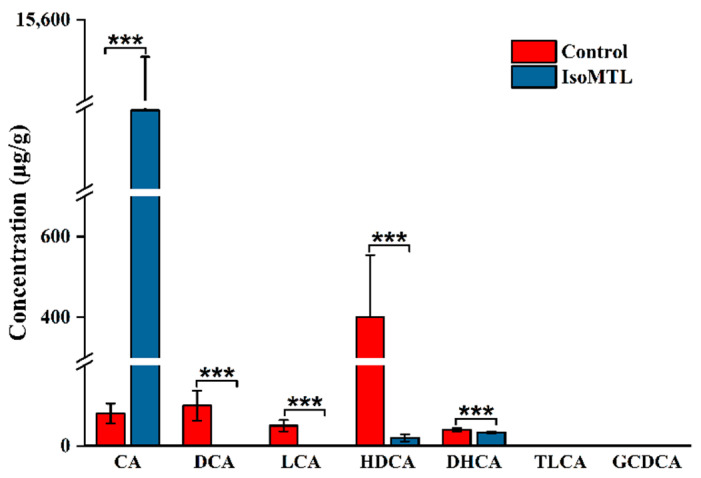
Dietary isomaltulose affects the concentrations of SBAs in fecal samples. CA, DCA, LCA, HDCA, DHCA, TLCA, and GCDCA refer to cholic acid, deoxycholic acid, lithocholic acid, hyodeoxycholic acid, dehydrocholic acid, taurolithocholic acid, and glycochenodeoxycholic acid. * Represents the differences between control and IsoMTL rats (*** *p* < 0.001).

Bile acid metabolism is closely associated with the development of gastrointestinal and metabolic diseases [24]. In the present study, the relative abundance of bile acid metabolism (secondary bile acid biosynthesis) was notably different in the rats fed with or without isomaltulose. To detect the changes in bile acid species, we performed a targeted metabolomic profiling using LC-MS. As shown in Figure 8, the concentration of cholic acid (CA) was notably higher, whereas the levels of deoxycholic acid (DCA), lithocholic acid (LCA), hyodeoxycholic acid (HDCA), and dehydrocholic acid (DHCA) were decreased in the IsoMTL group than the control group (*p* < 0.01). Moreover, the multiples changed in CA, DCA, LCA, HDCA and DHCA were 137.43, 129.51, 69.07, 19.86, and 1.44, respectively. In addition, the absence of taurolithocholic acid (TLCA) and glycochenodeoxycholic acid (GCDCA) was also observed in all feces samples. Therefore, these results demonstrated that dietary isomaltulose regulated bile acid metabolism and affected the composition and structure of bile acids.

### 2.6. Isomaltulose Affects the Fecal Levels of Short Chain Fatty Acids

**Figure 9:**
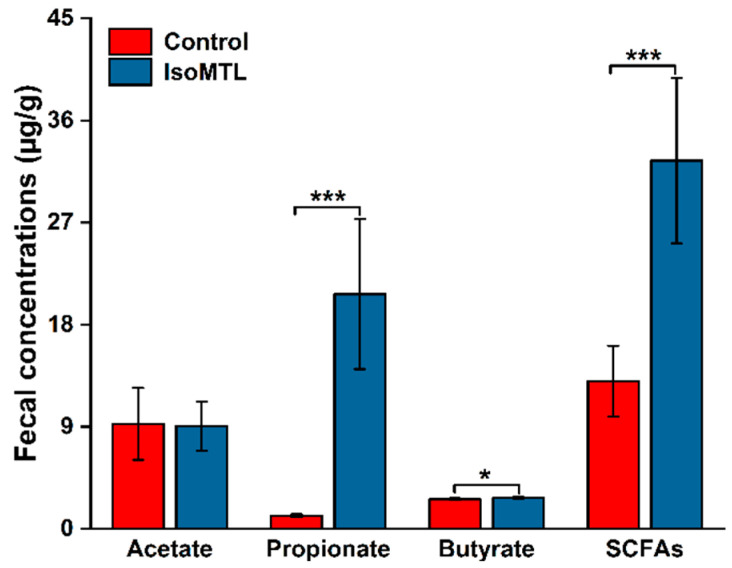
Dietary isomaltulose affects the production of SCFAs in fecal samples. The concentration of total short-chain fatty acids (SCFAs) refers to the sum of acetate, propionate, and butyrate. * represents the differences between control and IsoMTL rats (* *p* < 0.05, *** *p* < 0.001).

Short chain fatty acids (SCFAs) have been closely related to host physiology [2]. In the present study, several SCFAs-producing microbes were enriched in the rats fed with isomaltulose, which thus suggested that the SCFA composition and structure might be altered in fecal samples. Herein, a targeted quantitative analysis of SCFAs, acetic acid, propionic acid and butyric acid, was performed based on fecal samples through gas chromatograph (GC). As exhibited in Figure 9, the concentrations of propionic acid and butyric acid were elevated in the rats administered with isomaltulose in comparison with the control rats (*p* < 0.05). In particular, propionic acid was increased 18-fold by isomaltulose supplementation. Whereas, there was no substantial difference in acetic acid between the IsoMTL group and the control group. Moreover, the concentrations of total SCFAs (the sum of acetic acid, propionic acid, and butyric acid) revealed a significant improvement in the IsoMTL rats compared to the control group (Figure 9). Therefore, our data suggested that isomaltulose supplementation affected the levels of SCFA species.

## 3. Discussion

In the present study, we demonstrated that the consumption of isomaltulose could modulate the composition and structure of gut microbial populations. In particular, the abundance of beneficial microbes increased, whereas the level of conditioned pathogen decreased by isomaltulose supplementation. Moreover, we showed that isomaltulose ingestion affected bile acid metabolism and SCFAs metabolism in the intestines.

The rats fed with isomaltulose showed lower body weight than the control rats (*p* < 0.05, Figure 1A). Consistently, the food intake in the IsoMTL group was significantly decreased (*p* < 0.05, Figure 1B). In accordance with our results, a recent study also demonstrated that healthy overweight/obese subjects consumed the diet containing isomaltulose, which resulted in a profound weight loss and an obvious decrease in fat mass [25]. Another animal experiment also showed a notably lower body weight gain and food consumption rate when dietary food with isomaltulose [26]. Previous researches have reported that isomaltulose supplementation retards gastric emptying time, which leads to prolonged satiety and reduced energy intake [27]. Moreover, isomaltulose is a low Glycemic index (GI) ingredient, which triggers no significant increase in blood glucose, insulin, and HOMA-IR, thus consistent with our results. The oral glucose tolerance test exhibited that the consumption of isomaltulose contributed to promoting the recovery of blood glucose level in rats. In addition, the serum concentration of TG was significantly lower in the IsoMTL group than the control group (*p* < 0.05, Figure 1E), which was consistent with published human and animal experimental results [28,29]. These results suggest that ingestion of isomaltulose may have a potential metabolic benefit.

In the present study, the alpha-diversity indices, Chao, and Shannon index, were significantly reduced in the IsoMTL group when compared to the control group (*p* < 0.05, Figure 2A,B). Previous in vitro experiments indicated that isomaltulose might be a prebiotic [20,21]. Moreover, prebiotic is described as a substrate that is selectively utilized by the selected host microorganisms conferring healthy benefit [6]. Herein, we could hypothesize that the reduction in alpha diversity is related to the selectively stimulating effects of isomaltulose on several microbial communities. This hypothesis may be supported by recent reports, where ingestion of prebiotics. Inulin, FOS, GOS, cellulose, and xylooligosaccharides (XOS) also resulted in a lowered alpha-diversity [30,31]. PCoA score plot (beta-diversity) accounts for the similarity in microbial community [32]. Here, all samples were clustered into two groups, namely IsoMTL group and control group, which indicated that there was a substantial difference in bacterial similarity between the rats fed with and without isomaltulose (Figure 2C,D). Therefore, our data demonstrated that isomaltulose affected the diversity of gut microbiome.

To further identify the specific effects of isomaltulose on microbial species, we performed the LEfSe analysis based on the 16S rRNA sequencing data from phylum to genus. At the phylum level, the consumption of isomaltulose elevated the abundance of *Actinobacteria*, whereas reduced the level of bacterial phyla *Patescibacteria* (Figure 5). *Actinobacteria* is one of the four predominant intestinal bacterial phyla, which mainly includes three anaerobe genera *Bifidobacteria*, *Propionibacteria, Corynebacteria,* and aerobe genera *Streptomyces*. Previous studies have demonstrated that the phyla *Actinobacteria*, especially *Bifidobacteria*, can produce the glycosyl hydrolases, and hydrolyze the glycosidic bond within disaccharides and polysaccharides, such as FOS, GOS, XOS, inulin, and arabinoxylans [33,34,35]. By which, the species from *Actinobacteria* are supported to grow and proliferate. Isomaltulose is a naturally occurred disaccharide comprised of glucose and fructose linked by alpha-1,6 glycosidic bond. It can thus be hydrolyzed and utilized by *Actinobacteria* microbiota. Moreover, the species from *Actinobacteria*, i.e., *Bifidobacteria*, also metabolizes carbohydrates into SCFAs, mainly including acetic acid, propionic acid, and butyric acid [36]. SCFAs, besides as the primary energy sources for gut epithelial cells, contribute to the maintenance of intestinal homeostasis, barrier, immunology and host metabolism [36]. In addition, *Streptomyces* and other filamentous *Actinobacteria* have anti-tumor effects by producing antibiotics and thus damaging the DNA structure and functions of various tumor cells [37]. The decreased phyla *Patescibacteria* is likely an opportunistic or saprophytic colonizer. A previous study showed that *Patescibacteria* was significantly enriched in the group with alcoholic liver disease compared to the control, treatment, and positive groups [38]. Another research study also reported that the abundance of *Patescibacteria* was elevated in the signet-ring cell carcinoma patients [39].

Meanwhile, the ingestion of isomaltulose altered the abundance of eighteen bacterial genera (*p* < 0.05, Figure 5). Among these microbes, the genera *Faecalibacterium*, *Blautia*, *Phascolarctobacterium*, *Collinsella*, *UBA1819*, *Flavonifractor,* and *Holdemania* were significantly enriched in the IsoMTL group. The elevated genera *Faecalibacterium*, *Blautia*, *Phascolarctobacterium,* and *Flavonifractor* are the butyrate-producing bacteria, thus indicating that the consumption of isomaltulose could increase the concentration of butyrate [40,41,42]. This was confirmed by our targeted quantitative analysis of SCFAs, where the level of butyric acid showed a significant improvement in the rats fed with isomaltulose when compared to the control rats (*p* < 0.05, Figure 9). Butyrate has a crucial effect on host physiology and wellness. In addition to providing energy for the colonocytes, butyrate can suppress NF-κB transcription factor activation and interferon gamma and upregulate PPARγ, which thus withstand colorectal cancer and inflammatory bowel diseases [43,44,45,46,47]. Besides, the species *Faecalibacterium prausnitzii* from the genera *Faecalibacterium* has been also demonstrated to increase the tight junction protein expression, which, thus, affects paracellular permeability and maintains gut barrier function [48,49]. Succinate is a crucial substrate for the proliferation of *Clostridioides difficile,* which causes severe diarrhea. A recent study demonstrated that the *Phascolarctobacterium* species completely consumed succinate and thus inhibited the growth of *C. difficile* [50]. The species *Flavonifractor plautii* is a gram-positive anaerobic bacterium from the genera *Flavonifractor* belonging to *Firmicutes*. An animal experiment demonstrated that oral intervention of *Flavonifractor plautii* ameliorated antigen-induced Th2 immune responses and allergy by inhibiting interleukin-4 and IgE production and augmenting CD4^+^CD25^+^ T cells and CD103^+^CD11c^+^ DCs [51]. Another in vitro and in vivo study showed that the viable and heat-killed *Flavonifractor plautii* reduced inflammatory responses by suppressing the expression of proinflammatory cytokine TNF-α [52]. It is well known that isomaltulose has a tooth-friendly property. The mechanisms may be the structure of isomaltulose comprising glucose and fructose monomers linked by the stable α-1,6 glycosidic bond, which prevent the fermentation and acid production by oral bacteria [53]. *Shuttleworthia* is the normal component of oral microbiome. *Shuttleworthia satelles* belonging to the genera *Shuttleworthia* was isolated from infected root canals of teeth with endodontic abscesses, and closely related to the occurrence and development of periodontal disease [54,55]. This may be supported by a recent clinical trial, where the species *Shuttleworthia satelles* was enriched in the preschool children with caries [56]. Therefore, we speculate that the consumption of isomaltulose prevents cavities likely through the inhabitation of the growth of oral *Shuttleworthia satelles*. Overall, isomaltulose (e.g., FOS, GOS, and inulin) selectively promotes the growth of beneficial microbes, and inhibits the proliferation of pathogens.

The functional prediction analysis based on 16S rRNA sequencing data showed that the consumption of isomaltulose has a wide effect on biological functions in rats (Figure 7 and Figure 8). In our study, the abundance of carbohydrate metabolism, including starch and sucrose metabolism, galactose metabolism, fructose and mannose metabolism, and amino sugar and nucleotide sugar metabolism, was enhanced by ingestion of isomaltulose. This indicated that the enriched intestinal bacteria contributed to fermenting and utilizing dietary fiber, thus improving the bioavailability and absorption of minerals in the intestines [57]. There was a significant decrease in arachidonic acid metabolism in the rats fed with isomaltulose. Previous studies have demonstrated that arachidonic acid is metabolized into prostaglandins, thromboxane, and leukotrienes by cyclooxygenase, lipoxygenase, and cytochrome P450, respectively [58]. These metabolites of arachidonic acid provoke inflammatory responses [59]. Moreover, the level of energy metabolism, such as oxidative phosphorylation, was significantly enriched in the IsoMTL group. Oxidative phosphorylation has a close relationship to the energy supply through lipid and carbohydrate metabolism. Thus, isomaltulose ingestion might promote fat oxidation and energy efficiency. This could be supported by decreased body weight and food intake.

The abundance of bile acid metabolism, mainly secondary bile acid biosynthesis, was elevated by the consumption of isomaltulose. Our targeted metabolomic profiling showed that dietary isomaltulose enhanced the concentration of CA, whereas reduced the levels of DCA, HDCA, LCA, and DHCA (*p* < 0.05, Figure 8). CA is one crucial component of bile acids, which have a substantial effect on cholesterol homeostasis and participates in various metabolic processes as signaling molecules, i.e., lipid metabolism, cardiac, and gastrointestinal functions. Previous studies showed that CA promoted the reduction of cholesterol [60,61]. This was also in accordance with our results, where the level of TG was significantly lowered in the IsoMTL group (*p* < 0.05, Figure 2E). It is well known that CA is catalyzed into DCA and LCA by 7α-dehydroxylation from anaerobic microbiota, such as *Clostridium* [24,62]. Moreover, 7α-dehydroxylation is the strictly limited enzyme in the conversion of CA to DCA and LCA. In the present study, there was a significant decrease in the abundance of Clostridium species. This resulted in a reduction in 7α-dehydroxylation and thus in DCA and LCA. DCA and LCA can be modified into DHCA via several gut microbial enzymes. The decreased DCA and LCA further led to lessening DHCA. Herein, the consumption of isomaltulose regulates bile acid metabolism, likely related to the modulation of gut microbiota.

Mounting evidences have shown that SCFAs have a pivotal role in the homeostasis, barrier, inflammatory responses and epithelial integrity in the intestines [63]. SCFAs are the crucial metabolites of gut microbiota, and conversely modulate the composition of intestinal bacteria [2]. In the present study, the SCFAs-producing bacteria, including *Faecalibacterium*, *Blautia,* and *Phascolarctobacterium*, were notably enriched in the rats fed with isomaltulose. This may lead to the elevation of SCFAs. Consistently, our study exhibited a significant increase in total SCFAs, propionate, and butyrate in the IsoMTL group by GC profiling (*p* < 0.05, Figure 9). This was also supported by previously published in vitro studies, where propionate and butyrate were elevated when co-culture of microbial species with isomaltulose [21,22]. Additionally, according to functional prediction analysis based on 16S rRNA sequencing data, the abundance of alanine, aspartate, and glutamate metabolism was higher in the rats administered with isomaltulose. In the metabolic pathway, alanine is converted into pyruvate through the deaminization. Pyruvate can be metabolized to propionate through the succinate pathway or the acrylate pathway [2]. It is likely persuasive that *Phascolarctobacterium* species can produce propionate via the succinate pathway. In addition, pyruvate is also converted to butyrate by beta-hydroxybutyryl-CoA and crotonyl-CoA [2]. Therefore, the administration of isomaltulose enhances SCFAs-producing microbiota and thus the level of SCFAs.

## 4. Materials and Methods

### 4.1. Materials and Regents

All standards (acetic acid, propionic acid, butyric acid, cholic acid (CA), deoxycholic acid (DCA), lithocholic acid (LCA), hyodeoxycholic acid (HDCA), dehydrocholic acid (DHCA), taurolithocholic acid (TLCA), and glycochenodeoxycholic acid (GCDCA)) were purchased from Aladdin Biochemical Technology Co., Ltd. (Shanghai, China). Formic acid, methanol, sulfuric acid, diethyl ether, ammonium acetate, and acetonitrile (HPLC grade) were obtained from McLean Biochemical Technology Co., Ltd. (Shanghai, China). Isomaltulose was provided by BENEO GmbH (Mannheim, Germany).

### 4.2. Animal Experiments

Animal experiments were performed according to protocols approved by the Institutional Animal Care and Use Committee of Sun Yat-sen University (C2020-0330DS). The Laboratory Animal Center of Sun Yat-Sen university (Permission No: SCXK (Yue) 2016-0029) provided twelve male Sprague–Dawley rats, all five weeks of age. Experiments were carried out under SPF conditions with a 12/12 h light/dark cycle at 24 ± 2 °C and 50–70% humidity. Rats were randomly divided into two groups, namely control and isomaltulose (IsoMTL) groups (six rats in each group) after a seven-day acclimatization. Subsequently, the rats in IsoMTL groups were given free access to the water with isomaltulose (10%, *w/w*) and food, and the rats in control groups were given normal water and food for five weeks. Other conditions in two groups were the same. During the entire experiment, we observed the growth condition of all rats each day. Additionally, we determined the 24 h food and water intake of all rats on day 33.

### 4.3. Oral Glucose Tolerance Test

Oral glucose tolerance test (OGTT) was performed accorded to a previous published paper on day 34. Each animal fasted for 12 h, and was orally administered sterilized glucose solution (2.0 g/kg, Sigma-Aldrich, Louis, MO, USA). Blood samples were collected from the tail vein at five time points (0, 30, 60, 90, and 120 min), and used to measure the glucose concentrations through a glucose-meter (ONETOUCH Ultra, LifeScan, Milpitas, CA, USA).

### 4.4. Sample Collection

At the end of the experiment, stool samples of each rat were carefully collected in microcentrifuge tubes, and immediately frozen in −80 °C for microbiome profiling and targeted quantitative analysis of SCFAs and SBAs. Blood samples were also cautiously collected from the abdominal aorta and centrifuged at 3000 rpm for 20 min at 4 °C to obtain serum, which was then used for biochemical assays. After the rats were sacrificed, colon and hepatic tissues from all animals were collected for histological analysis.

### 4.5. Histological Analysis

Hepatic and distal colonic tissues were fixed with 4% paraformaldehyde buffer for 48 h, dehydrated, sectioned at 3 µm thickness, and then stained with hematoxylin and eosin (H&E). Subsequently, colonic epithelial and crypt integrity, cell infiltration and mucosal thickness, and the cell integrity, size, and arrangement of liver tissues, hepatic steatosis, and the size and density of hepatic fat vacuoles, were analyzed by a digital image analysis system (Leica DM5000B microscope, Wetzlar, Germany).

### 4.6. Biochemical Assays

Fasting blood glucose (FBG) was detected by touch Ultra glucometer (LifeScan, Milpitas, CA, USA). We measured the serum concentrations of fasting serum insulin (FSIns), lipopolysaccharides (LPS), triglyceride (TG), low-density lipoprotein (LDL), and total cholesterol (TCHO) by ELISA, using specific kits. Briefly, all serum samples were first taken from −80 °C, thawed at 4 °C, and then treated in accordance with the instructions of each ELISA kit (Maisha, China). OD values were determined at 450 nm using a microplate reader (FlexStation 3, Molecular Devices, FL, USA).

### 4.7. Fecal DNA Extraction

Stool sample DNA was extracted using a NucleoSpin 96 Soil Kit (MN, Germany) according to the manufacturer’s instructions. A Bioanalyzer 2000 (Agilent, Palp Alto, CA, USA) was employed to verify DNA integrity and a Qubit^TM^ fluorometer (Invitrogen, Corp., Carlsbad, CA, USA) was used to measure DNA concentration.

### 4.8. 16S rRNA Gene Sequencing and Analysis

The hypervariable V3-V4 region of the 16S rRNA genes of fecal bacteria was sequenced using primers 338F (5′-ACTCCTACGGGAGGCAGCA-3′) and 806R (5′-GGACTACHVGGGTWTCTAAT-3′) on the Illumina HiSeq 2500 PE250 platform (Illumina, Inc. Common, CA, USA). Data preprocessing of the reads obtained was carried out as follows: (a) FLASH v1.2.7 software was used to merge overlapping reads to obtain merged raw tags; (b) Trimmomatic v0.33 software was used to filter raw tags to acquire high-quality clean tags; (c) UCHIME v4.2 software was employed to identify and remove chimeric sequences to obtain effective tags. Data quality was assessed by evaluating sequence number, sequence length, CG content, Q20, and Q30 quality values and effectiveness at each stage of data processing (Table S2). To facilitate further analysis, sequences with similarity ≥97% were considered an OTU (Operational Taxonomic Unit). USEARCH software was subsequently employed to carry out taxonomic annotation of OTUs based on the Silva (Bacteria) and UNITE (fungi) taxonomic databases. To obtain the corresponding species classification information, all OTUs were aligned to microbial reference databases from phylum to species. Subsequently, QIIME software was used to generate species richness at every taxonomic level. OTUs with low richness ≤0.005% were filtered and the final OTU list is presented in Table S2. Next, mothur software v.1.30 was used to evaluate the Alpha diversity index and species richness of samples using Chao1, Shannon and rank abundance curves. QIIME software was used to analyze Beta diversity (principal coordinate analysis, PCoA) by unweighted UniFrac methods. To further understand whether the altered intestinal microbial composition might affect metabolism, PICRUSt software was employed to predict functional gene composition and the associated metabolic pathways by comparing species composition information from 16S sequencing data with KEGG pathways. STAMP was used for functional profiling.

### 4.9. Quantitative Metabolomics Profiling of Fecal SCFAs

A total of 200 mg stool samples were taken from −80 °C, thawed at 4 °C, and vortexed with 1000 µL distilled water for 10 min, and centrifuged at 5000 rpm at 4 °C for 10 min. Subsequently, 500 µL of the supernatant was filtrated using 0.22 µm Millipore filter, thoroughly mixed with 50 µL of 50% sulfuric acid solution (*v/v*). Then, 800 µL diethyl ether was used for SCFAs extraction for 20 min and centrifuged at 10,000 rpm at 4 °C. The supernatant was taken for SCFAs determination by gas chromatograph (GC, 7890B CG System, Agilent Technologies Corporation, CA, USA) equipped with DB-FFAP (0.25 µm × 0.32 mm × 30 m). Moreover, 2-ethyl butyric acid was used as the internal standard.

The main parameters were set as follows: (a) the carrier gas was nitrogen gas at a rate of 2.0 mL/min; (b) the injection volume was 1.0 µL; (c) the injection and ionization temperatures were 230 and 250 °C, respectively; (d) the gradient conditions were set as the initial temperature of 100 °C for 0.5 min, rose to 170 °C at a rate of 8 °C/min and maintained for 0.5 min, and then rose to 220 °C at a rate of 20 °C/min and maintained for 2 min.

### 4.10. Quantitative Metabolomics Profiling of Fecal SBAs

A total of 40 mg of stool samples were taken from −80 °C, thawed at 4 °C, and vortexed with 350 µL methanol aqueous solution (50%, *v/v*) and 10 µL internal standard (GCDCA-d4) for 5 min, and then centrifuged for 10 min at 13,000 rpm at 4 °C. The supernatant was centrifuged for 5 min at 13,000 rpm at 4 °C. Then the supernatant was taken for SBAs analysis through high performance liquid phase tandem mass spectrometry (LC-MS-7080 system, Shimadzu Corporation, Japan).

The chromatograph column is ACQUITY UPLC HSS T3 (1.8 µm × 2.1 mm × 100 mm, Waters Crop, Milford, MA, USA). The mobile phase A and B were 0.1% formic acid water (*v/v*) mixed with 5 mM ammonium acetate and acetonitrile, respectively. The basic parameters were set as follows: the column temperature: 35 °C; the flow rate of mobile phase: 0.35 mL/min. The gradient conditions were as follows: 5% A at 0 min; 5% A at 0.5 min; 10% A at 1.5 min; 85% A at 3 min; 98% A at 6 min; 5% A at 6.1 min.

The main parameters of mass spectrometry were set as follows: the flow rate of atomizing, heating, and drying gas were 3, 10, and 10 L/min, respectively. The interface, desolvation, and heating temperatures were 300, 250, and 400 °C, respectively.

### 4.11. Statistical Analysis

The difference was compared by one-way analysis of variance (ANOVA) with Tukey–Kramer post hoc method using SPSS v26.0 (Chicago, IL, USA). Data visualization was performed by R language packages, O-Ring Pro 2020 (OriginLab, MA, USA), and the Omicsolution platform (https://www.omicsolution.org/wkomics/main/, Accessed date on 20 Feb 2021). All data were shown as the mean ± standard deviations.

## 5. Conclusions

In summary, the present study demonstrated that consumption of isomaltulose modulates the composition of gut microbiota, and the production of SCFAs and SBAs in rats. Beneficial microbes are enriched in the rats administered with isomaltulose, whereas opportunistic pathogens are suppressed. Moreover, isomaltulose ingestion notably affects functionalities of gut microbiota, including the elevation of alanine, aspartate and glutamate metabolism, SBA biosynthesis, and the inhibition of arachidonic acid metabolism. In accordance with the results of gut microbiota, isomaltulose supplementation enhances the concentration of CA, and reduces the levels of DCA, LCA, DHCA, and HDCA. Moreover, the concentrations of propionate and butyrate are significantly improved in the rats consuming isomaltulose. Therefore, this work suggests the modulation of isomaltulose on gut microbiota, and may provide a theoretical basis for its use in improving host health as a prebiotic.

## Data Availability

The data presented in this study are available from the corresponding author.

## Supplementary Materials

The following are available online. Figure S1: The effect of isomaltulose onglycolipid metabolism in rats. Figure S2: The effect of isomaltulose on Serum level of lipopolysaccharide (LPS) in rats. Figure S3: The effect of isomaltulose on the histopathology of liver and colon tissues. Table S1: The functional composition of gut microbiota predicted by PICRUSt2 using 16S rRNA sequencing data.

## Sample Availability

Samples of isomaltulose are available from the authors.
