# Supplementary material for: Isomaltulose Exhibits Prebiotic Activity, and Modulates Gut Microbiota, the Production of Short Chain Fatty Acids, and Secondary Bile Acids in Rats
Source: Molecules. 2021 Apr 23;26(9):2464. doi: 10.3390/molecules26092464 (PMC8122910; doi:10.3390/molecules26092464)
Supplement: Supplementary file 1 [file molecules-26-02464-s001.zip › Supplementary Figure.pdf]

Supplementary Figures

Isomaltulose Exhibits Prebiotic Activity, and Modulates Gut Microbiota, the Production of Short Chain Fatty Acids, and Secondary Bile Acids in Rats

Zhan-Dong Yang [1], Yi-Shan Guo [2], Jun-Sheng Huang [2], Ya-Fei Gao [2], Fei Peng [3], Ri-Yi Xu [2], Hui-Hui Su [2,*] and Ping-Jun Zhang [2,*]

[1] School of Pharmaceutical Sciences, Sun Yat-sen University, Guangzhou 510006, China; yangzhd5@mail2.sysu.edu.cn (Z.-D.Y.)

[2] Guangdong Engineering Lab of High Value Utilization of Biomass, Institute of Bioengineering, Guangdong Academy of Sciences, Guangzhou 510316, China; yishan.guo@foxmail.com (Y.-S.G.); huangjunsheng2020@foxmail.com (J.-S.H.); csicgao@foxmail.com (Y.-F.G.); scutriyal@iCloud.com (R.-Y.X.)

[3] School of Food Science and Engineering, Nanchang University, Nanchang 330000, China; pengf0129@foxmail.com (F. P)

* Correspondence: fesuhuihui@mail.scut.edu.cn (H.-H.S.); zhangpingjun886@sina.com (P.-J.Z.); Tel.: +86-020-8416-8316 (H.-H.S.)

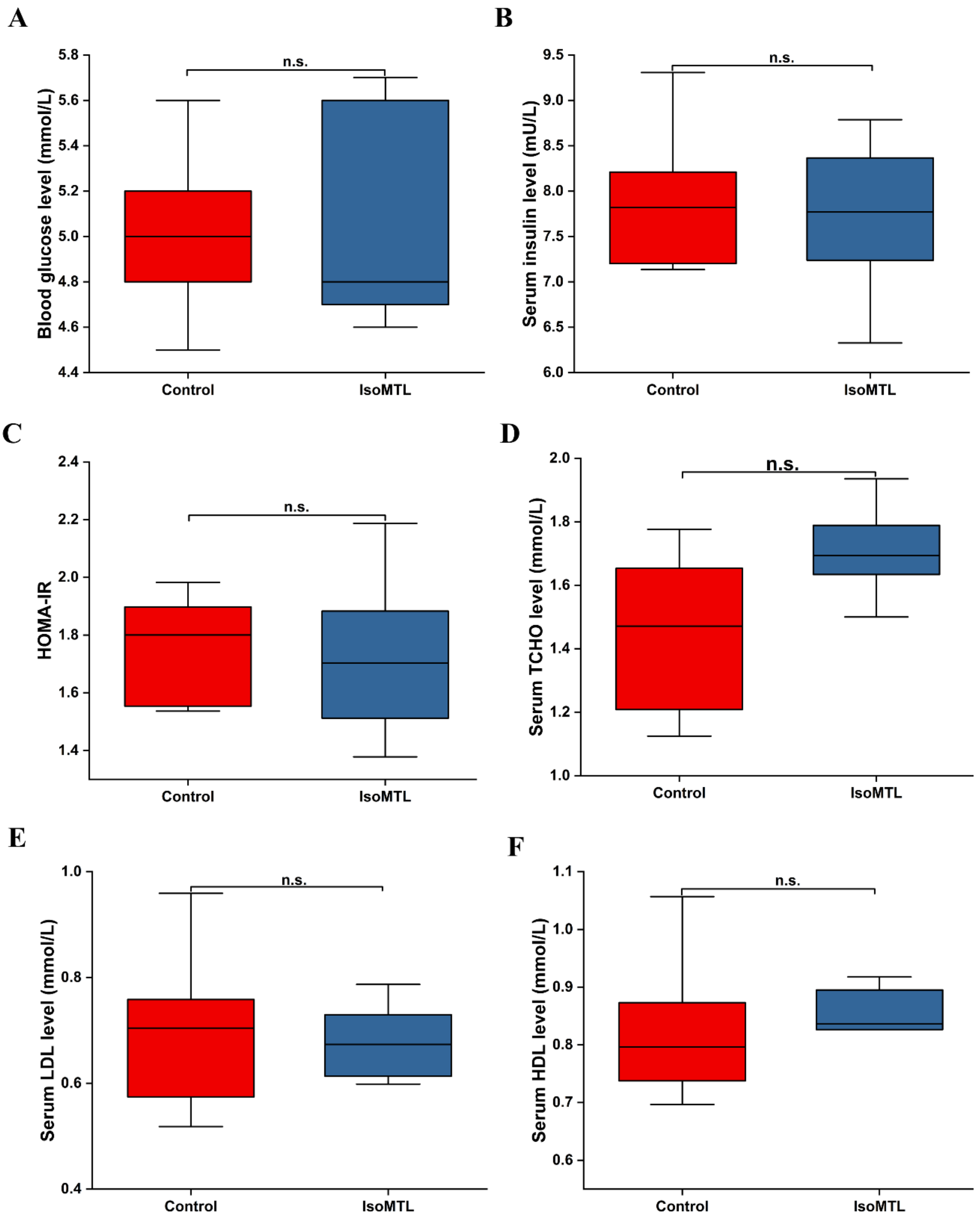

Supplementary Figure S1. The effect of isomaltulose on glucolipid metabolism in rats, including (**A**) Blood glucose level (FBG), (**B**) serum insulin level (FSIns), (**C**) HOMA-IR (homeostasis model assessment of insulin resistance), (**D**) serum total cholesterol (TCHO) level, (**E**) low-density lipoprotein (LDL) level and (**F**) high-density lipoprotein (HDL) level. n.s. refers no significant difference between two groups.

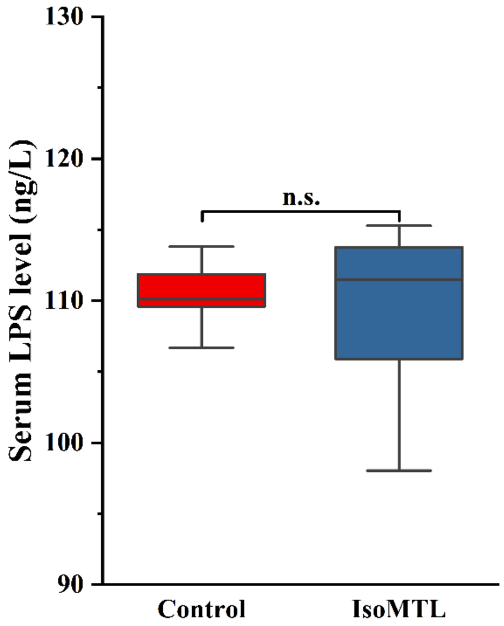

Supplementary Figure S2. The effect of isomaltulose on Serum level of lipopolysaccharide (LPS) in rats. n.s. refers no significant difference between two groups.

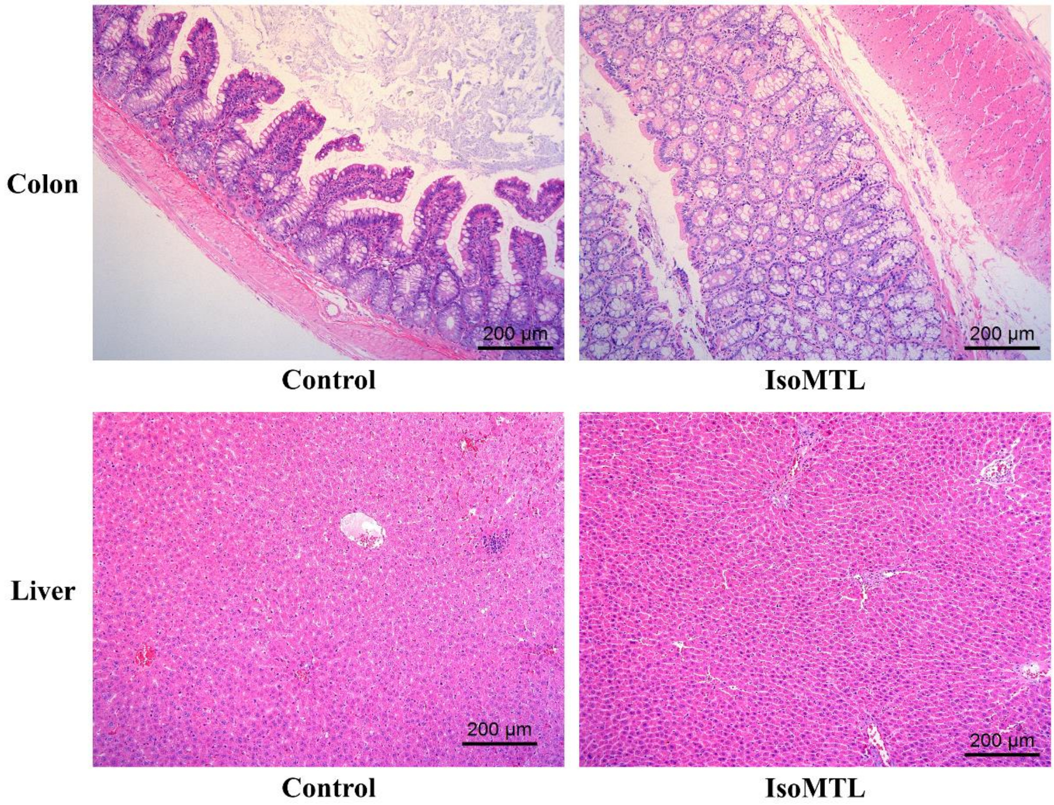

Supplementary Figure S3. The effect of isomaltulose on the histopathology of liver and colon.
